# Social networks and expertise development for Australian breast radiologists

**DOI:** 10.1186/s12913-016-1938-9

**Published:** 2017-02-11

**Authors:** Seyedamir Tavakoli Taba, Liaquat Hossain, Karen Willis, Sarah Lewis

**Affiliations:** 1 Complex Systems Research Group, Faculty of Engineering & IT, University of Sydney, Sydney, Australia; 2 Division of Information & Technology Studies, Faculty of Education, University of Hong Kong, Pok Fu Lam, Hong Kong; 3 Faculty of Health Sciences, Australian Catholic University, Melbourne, Australia; 4 Medical Imaging Optimisation and Perception Group (MIOPeG), Faculty of Health Sciences, Brain Mind Research Institute, University of Sydney, Sydney, Australia

**Keywords:** Social Networks, Expertise Development, Performance, Experience, Feedback, Learning, ICT, Radiology

## Abstract

**Background:**

In this study, we explore the nexus between social networks and expertise development of Australian breast radiologists. Background literature has shown that a lack of appropriate social networks and interaction among certain professional group(s) may be an obstacle for knowledge acquisition, information flow and expertise sharing. To date there have not been any systematic studies investigating how social networks and expertise development are interconnected and whether this leads to improved performance for breast radiologists.

**Methods:**

This study explores the value of social networks in building expertise alongside with other constructs of performance for the Australian radiology workforce using semi-structured in-depth interviews with 17 breast radiologists.

**Results:**

The findings from this study emphasise the influences of knowledge transfer and learning through social networks and interactions as well as knowledge acquisition and development through experience and feedback. The results also show that accessibility to learning resources and a variety of timely feedback on performance through the information and communication technologies (ICT) is likely to facilitate improved performance and build social support.

**Conclusions:**

We argue that radiologists’ and, in particular, breast radiologists’ work performance, needs to be explored not only through individual numerical characteristics but also by analysing the social context and peer support networks in which they operate and we identify multidisciplinary care as a core entity of social learning.

**Electronic supplementary material:**

The online version of this article (doi:10.1186/s12913-016-1938-9) contains supplementary material, which is available to authorized users.

## Background

Breast radiologists are expert radiologists who often work in isolation or make blinded decisions on pathology such as cancer. In this study, we explore expertise development, knowledge sharing and social networks in the Australian radiology workforce and links to improved performance of Australian breast radiologists.

The ability to solve complex problems by utilising new information and developing in-house knowledge is an important feature of job performance in knowledge intensive works, such as breast radiology. Many conventional definitions and theories of job performance focus solely on individual attributes and characteristics such as ability, knowledge, skill, experience, personal attitudes and self-esteem [1–5]. While the effects of individual characteristics on job performance is acknowledged, some studies investigating the performance dynamics of knowledge intensive workers claim that social networks facilitating connectedness among workers significantly influence their job performance [6–8]. We thus draw on social networks theories to explore the nexus between social networks and expertise development in Australian radiology.

## Introduction

### Radiology and expertise

Radiology services have a major role in current diagnostic health care and there has been a significant increase in medical imaging services within the past 20 years, resulting in pressure on the current available workforce [9, 10]. Classified among the best in the world, Australia has a complex health system that incorporates both public and private sectors. Expenditure on diagnostic imaging (primarily radiology) is estimated to be 15% of all Medicare (Australia’s public and primary funder of health care) expenses in 2014–15 [11]. A number of social and economic factors such as an ageing population, increasing awareness around health and wellness, medical payment models (for example, the Medicare Benefits Schedule in Australia) have contributed to the raised radiology load without an associated rise in the number of radiologists performing this work.

Previous studies have identified imbalances between the supply and demand of the Australian radiology workforce [12–15]. It has been suggested that the existing models of radiological services may be unsustainable and unable to meet increasing demands [16, 17] and further, there is a need to broaden or deepen the radiology knowledge system and improve radiologists’ performance to meet future demand for high quality medical imaging services. Thus, it is important to understand different constructs of job performance and the processes by which knowledge development occurs within the radiology profession.

Image interpretation is a core skill of radiologists and this is often termed “observer performance” in radiology professional literature [18, 19]. BreastScreen Australia is a national program offering a mammographic breast cancer screening service for Australian women aged 50–74 years. BreastScreen radiologists carry out screen reading (reading sessions) and/or diagnostic procedures (such as assessment clinics for further imaging and biopsies for women who are recalled). The mammograms are read by two BreastScreen radiologists in a double blind mode and if both reads are positive, the woman will be referred for assessment. When there is a disagreement between two primary reads (one positive and one negative), a third adjudicated screen reader will read the case and decide the outcome. Mammographic interpretation in a population screening environment, such as BreastScreen Australia, is one of the most challenging tasks in the radiology profession with a comparatively high error rate but with a binary decision of abnormal (the likely presence of malignancy or breast cancer) and normal (no malignant disease suspected) [20].

The cognitive study of expertise in knowledge works provides valuable information about underlying personal cognitive processes involved in performance improvement. V Patel, R Glaser and JF Arocha [21] provide an overview of the characteristics of expertise regarding acquisition of medical competence and superior performance. They argue that high performance is associated with structured and interconnected domain-specific knowledge. The performance becomes gradually superior and efficient when practitioners gain experience in the execution of a task. Until the knowledge becomes completely consolidated, practitioners are more likely engaged in the search of unnecessary and irrelevant information. However, by experience, experts gain the competence to fine tune their knowledge to perform their tasks more effectively.

Previous studies show that observer performance in mammography is primarily associated with individual characteristics, such as years certified as a radiologist, years reading mammograms, number of mammograms read per year and hours reading mammograms per week [22–24]. However, there is no available research examining the effects of social networks on the performance of radiologists, in either general radiology or mammography. Hence our study investigated the additional construct of social networks and expertise development alongside more traditional individual constructs such as experience.

### Social networks and job performance

The social networks of professionals often affect job performance by providing them with new critical perspectives of knowledge and information [6]. Some social networks theories, such as structural holes [25], explain the variability of adaption and learning among actors (workers) of a particular network and the level of new information and knowledge that they have access to. Structural holes, in a social network, are the non-existent links among actors or clusters/groups in the network, which can potentially be connected together. According to structural holes theory, actors who tie these holes have a privilege in access to information and knowledge which others don’t have and consequently, bridging structural holes is positively associated with performance in knowledge works [26, 27].

On the other hand, actors who lack appropriate peer support networks or actors who interact only within a bonded network may not have access to new information; therefore the information they communicate is redundant. While some studies show that individuals with higher numbers of contacts (known as their degree centrality) tend to show higher levels of performance [3], RS Burt [25] asserts that if the number of direct contacts is not coordinated with the diversity of contacts reached by the actor, it may result in increasing the number of structural holes and ultimately decreasing efficiency in the network. In this regard, efficiency refers to reaching to the maximum number of contacts through direct and indirect relations, using the minimum primary contacts. Previous researchers showed that network constraint (as opposed to efficiency) limits actors’ novelty because actors in such a network are not likely to receive new and diverse information and so, network constraint decreases abilities and performance proficiencies of such actors [28].

The extent of bonding of actors within a network also depends on the strength of their ties (frequency and/or closeness of relationships) with each other. The strength of weak ties theory [29] asserts that information is circulated more quickly in strongly bonded clusters than the rest of network but this information usually becomes redundant in a relatively short time. Studies by D Krackhardt [30] and DZ Levin and R Cross [31] suggest that strong ties facilitate trust building and acquisition of knowledge, which longitudinally improve learning and job performance levels. Information and communication technologies (ICT) also affect collaboration and learning process in knowledge intensive works. Recent developments in ICT have advanced collaborative activities in virtual settings and ICT provides new alternatives for many traditional face-to-face social interactions [32]. In the context of medical departments, DL Paul [33] argue that ICT can considerably enlarge the knowledge resources offered to medical practitioners, but may also create additional challenges to collaboration activities in some cases.

In view of this evidence, we believe that the interconnectedness of social networks, individual characteristics and ICT use all need to be considered when one explores job performance in knowledge workers. Using theory from KSK Chung and L Hossain [7], we began this study with a preliminary framework that conceptualises how performance in knowledge intensive fields is achieved. Our framework includes social networks, individual characteristics, and the level of ICT use. This framework forms the basis for our investigation exploring the interconnectedness of social networks and expertise development for the case of Australian Breast Radiologists.

## Methods

As this is a novel exploration of expertise development in radiology, a qualitative approach was considered best to document radiologists’ experiences, ideas and perceptions. Qualitative research can provide researchers with rich, real world data which can be used to achieve new insights [34] from radiologists’ point of view about their performance constructs. The qualitative study sought to investigate the relative strengths and connections between expertise development and social learning. We designed a semi-structured interview study with radiologists to cover three main topics: the influence of social networks in knowledge transfer and expertise sharing among the radiology workforce; radiologists’ individual work habits in reporting as well as knowledge acquisition and expertise development contextualised to breast radiology; and the application of ICT in different aspects of their professional career. In addition to the semi-structured questions, prompts were used to elicit deeper information on these topics (see Additional file 1: Appendix for the list of interview questions).

Qualified radiologists who regularly report on breast images were invited to participate in semi-structured interviews. Ethics approval was received from the University of Sydney Human Research Ethics Committee (Project No.: 2014/485). A participant recruitment flyer was distributed at a major radiology conference in 2014 and the interviews took place at the conference venue. Seventeen Australian breast imaging radiologists were recruited for the study. The length of the interviews ranged from 30 to 70 min. All interviews were audio recorded and transcribed in full.

Interviews were analysed using an inductive approach enabling identification of themes relating to knowledge acquisition, social networks and ICT use. The software program NVivo 10 was used to organise the data. The grounded theory method was carried out by firstly reviewing data collected, looking for key and repeated ideas and ‘open coding’ the concepts based on ‘constant comparative analysis’ across all transcripts [35]. The transcripts were coded by one researcher (ST) and then reviewed by another two researchers with expertise in qualitative analysis (SL, LH). The methodology used in this study adheres to RATS qualitative research review guidelines.

## Results

Most of the 17 Australian radiologists were highly experienced, with strong expertise in breast imaging and primarily working in breast image interpretation. Table 1 shows participants’ demographic and some personal characteristics. The sample is reflective of the gender mix in Australian radiology [17]. Participants had a variety of public and private sector jobs and most of them were also a BreastScreen reader.

**Table 1:** Participants’ demographics

Participant code	Age group	# Years certified as a radiologist	# Mammogram cases read per week (incl. diagnostic)
I01	36–45	≤5	51–100
I02	56–65	16–20	>200
I03	36–45	6–10	<20
I04	56–65	21–25	>200
I05	>65	>31	<20
I06	56–65	26–30	101–150
I07	46–55	16–20	<20
I08	56–65	>31	<20
I09	56–65	>31	101–150
I10	36–45	11–15	<20
I11	56–65	26–30	101–150
I12	56–65	26–30	>200
I13	>65	>31	<20
I14	36–45	6–10	<20
I15	46–55	21–25	>200
I16	36–45	≤5	51–100
I17	56–65	>31	101–150

Six key concepts were formed by combining connected themes which emerged from the interviews. The key concepts were *the value of communication*, *different patterns of social networks*, *the value of experience*, *feedback and expertise*, *essential radiology functions* and *social/transportable workplaces*. The sub concepts, which represent smaller distinct concept under a broader key concept, are discussed throughout the text. All categories of inquiry, key concepts and concepts are demonstrated in Table 2.

**Table 2:** Summary of inquiry, key concepts and concepts

Category	Key Concept	Concepts
Social Networks and Learning	The Value of Communication	Looking for Expertise and Validation
		Multidisciplinary Care
	Different Patterns of Social Networks	Workplace/Environment Influence
		Network Constraint
		Various Strength of Ties
Knowledge Acquisition and Development	The Value of Experience	Repeated Exposure
		Self-directed Education
		Systematic Searching
	Feedback and Expertise	General Feedback on Reported Cases
		BreastScreen Feedback Loop
Role of Information and Communication Technologies (ICT)	Essential Radiology Functions	Digital Libraries
	Social/Transportable Workplaces	

### Social networks and learning

To begin to explore how radiologists viewed social networks and learning, we asked them about how they search for expertise and feedback. The overwhelming responses relate to the value of communication with others as well as different patterns of social networks within, and external, to the radiology profession.

#### The value of communication

Social networking and communication with other radiologists and clinicians was a key theme and included concepts around radiologists seeking experts in their field for validation as well as appreciating the value of multidisciplinary care and communication.

##### Looking for expertise and validation

In every medical field, there are people recognised as having higher expertise in a particular specialty domain. Radiologists in this study, particularly those more experienced, identified that if they do not have a lot of expertise in a certain radiological domain, they will probably know somebody who has greater expertise and attempt to seek them out:

*Having access to people who are more expert than you [is important] to help resolve the difficult questions and help further your expertise. [I11]*

*It’s always useful to have someone’s advice who has a higher level of expertise in a particular area when we need it. [I03]*

According to radiologists, one dilemma within radiology is that they are in an environment where it is not immediately obvious whether their decision is correct or not as often reporting is done in isolation from any cytology verification. In this regard, radiologists also discussed improving their interpretation by seeking a second opinion from a colleague. They believed that it is always useful to discuss a difficult case with other radiologists even, as one radiologists stated, “*if sometimes they are not any more expert than you, to at least see if there is a match-up on something that’s a bit equivocal” [I01],* indicating that validation is an important reason for seeking expertise.

Some participants believed that social interactions within the assessment clinics or through attending external meetings, workshops and conferences are imperative for improving performance because a lot of required knowledge in either general or breast radiology is conveyed informally. As one participant stated:

*So a lot of it is done informally, the knowledge you gain, and also it’s done through meetings and presentations like this [venue of interview] … [So], I think they’re very important, I think being able to mix with your colleagues at conferences and other environments is pretty important. [I17]*

**Multidisciplinary care**

A further theme emerging from the interviews was that communication with referrers and external specialists, such as pathologists and breast surgeons, is critical for gaining new knowledge and improving performance. It was commented by the participants in this study that external clinicians provided radiologists with real feedback on their diagnoses and this, in turn, enhanced confidence. In particular, social interaction and discussions with pathologists and surgeons were found to enrich the feedback provided through pathology reports:

It has previously been reported that one important event where radiologists can meet other cancer care clinicians and discuss the diagnosis and management of patient cases are multi-disciplinary team (MDT) meetings [36]. According to our interviews, radiologists learned a lot from MDT meetings, specifically by seeing the results of the biopsies that they had done on lesions from the last week or month. During an MDT meeting, radiologists reported discussing why they believed the case was abnormal and then pathologists and surgeons may provide evidence as to whether the lesion is benign or malignant. This discussion and feedback from other clinicians was found to be very constructive and our findings concur with SB Alcantara, W Reed, K Willis, W Lee, P Brennan and S Lewis [36] regarding the benefits of MDT meetings. The frequency of MDT meetings vary from weekly to monthly in different practices and attendance is voluntary by radiologists and the time demands imposed to radiologists was known as a barrier for not attending [36].

*I can learn a lot because I’m doing the MDT meeting. At our MDT meetings, we’ve got pathologists that show us the slides, and we discuss the case. Firstly, the radiologist says this is suspicious, and the pathologist there suggests I confirm this is a cancer and also show us why it looks like a mass lesion, why it looks more like a distortion … Sometimes, we discuss difficult cases. And the feedback from the pathologist, what we call correlation is important. [I02]*

#### Different patterns of social networks

Radiologists described different configurations of social networks in their professional work group. The findings show the effects of workplace and environment on network patterns, network constraint in almost all radiology networks, and various strength of ties for different radiologists.

##### Workplace/environment influences

According to participants there was a good degree of difference in the networking patterns between different radiology workplaces and environments. In many public hospitals and private practices, it was reported to be very common that a radiologist would contact other specialists and referrers for supplementary information about their patients or requests and radiologists frequently ask each other for second opinions. Importantly, this was seen to be a cultural attribute of the workplace:

*In our department, because it’s a teaching hospital there’s a culture of if you’re not sure about something, you ask. So that’s happening all the time. So I think it’s important to have people that are available in the department to talk to, and there’s a culture of asking for help. [I12]*

However, in other environments, such as BreastScreen, radiologists undertake bulk reading often in isolation and there are not such opportunities for on the spot discussion and immediate feedback from colleagues:

*Actually, when we are reading, it’s different in the screening program than in the private [practice]. In the private, when radiologists don’t feel confident to say this mammogram is normal, they will ask one of the colleagues. In the BreastScreen, you can’t start to ask people because you are in your cubicle. You are by yourself. [I02]*

Yet, radiologists in this study that were readers for BreastScreen also noted that there was the opportunity to communicate with other radiologists and get feedback when they conduct assessment clinics. Assessment clinics are weekly follow up sessions for recalled women who require additional imaging, biopsy and tertiary consults and are a precursor to multidisciplinary care. One such reported case scenario by a participant is when radiologists are at the clinic and a difficult case presents, there is likely another radiologist, expert or similar, may call in to request a second opinion:

*[At BreastScreen], sometimes people call from another service and say look, I’m just at the clinic. Can you just have a look? … That means I can log in [to BIS], and tell them look, I think this is benign, or do an x-ray view or do this one. [I02]*

##### Network constraint

The interviews revealed that most of breast radiologists had very bonded social networks. While radiologists acknowledged the importance of job-related interactions, personal connections and immediate feedbacks from others, they stated that they rarely contacted people outside of the country or outside of their home state. More importantly, the majority of radiologists’ social-expertise contacts were found to be “*just in [their own] department” [I12]* and/or within “*the same company [practice]” [I03]* where they work.

Workplaces were found to affect the networking opportunities for breast radiologists. In most private radiology practices, where cases are commonly reported on the same day as the patient is imaged, radiologists reported that the amount of expert seeking behaviour is reduced. The information and communication systems available in different workplaces also impact the boundaries of a radiologist’s network. It may be that some radiological workflow systems may limit communication and image distribution within one physical workplace. In this case, radiologists stated that they may wish to contact people who they *“know and have worked with in the past, but it’s a little bit harder to do on a day-to-day basis because [they] can’t transfer the images instantaneously”. [I01]* However, some systems may be common or compatible across several sites and this facilitates radiologists to ask colleagues at other sites to have a look at an image for their opinion:

*Our messages are instantaneous … and you can distribute the images if you want a second opinion. So you can ask someone in a different branch. [I04]*

##### Various strength of ties

We asked participants how frequently they approach other radiologists or were being approached by radiology colleagues to discuss a difficult case or search for expertise. The responses were moderately diverse and frequency of communication ranged from “*only once a month” [I08]* to “*once or twice daily” [I01].* Most of responses, however, were somewhere between this range with *“half a dozen times a month” [I03]* more indicative of the frequency.

### Knowledge acquisition and development

Radiologists talked about knowledge acquisition and how they personally acquired expertise within the sometimes closed environment of a radiology department. The responses in this section relate to the value of experience as well as importance of feedback for the development of expertise.

#### The value of experience

Radiologists emphasised the value of experience as an important concept underpinning observer performance in breast radiology. They described that repeated exposure to mammograms, ongoing self-directed education, and systematic searching when reading images can all affect their performance.

##### Repeated exposure

Similar to other studies [20], radiologists in this study believed that reading large volumes of mammograms is essential for high performance. *“In Australia, you have to read at least 2,000 of mammography a year to become an expert”. [I09]* Radiologists linked reading of large batches of mammographic cases to enhanced performance. They believed that repeated exposure to a variety of appearances helped them to appreciate the subtleties of different forms of breast cancer:

*Number of images. That’s the bottom line. You’ve got to have the imprint in your brain with what the normal is and what the abnormal is. [I11]*

According to participants, one issue in training general radiology registrars in Australia is that they don’t see enough mammograms on a day-to-day basis, unless they rotate through BreastScreen. So in the setting of a public hospital system, registrars may get very sporadic training in breast imaging, which may be enough to *“pass the exams” [I17],* but does not equate to expertise or competency. Moreover, radiologists reflected that there are still work environments where registrars *“don’t get any exposure to breast imaging. So they’re not rotated through breast imaging at all, so they’re not trained. If you don’t have any exposure, you can’t read”. [I15]*

##### Self-directed education

In a clinical environment such as BreastScreen, small numbers of true-positive cases of cancers are mixed with large numbers of normal cases and screening is a binary decision (to recall or not to recall the woman). Radiology training often relies on enriched test sets or case sets for exposure to cancer appearances, but one of the key learning points as told by the radiologists in this study was about the importance of finding a cancer among a wide range of normal appearances. Breast radiology performance is intrinsically linked to specificity performance, which is the ability to identify normal cases and minimise false positive decisions. But “*one thing that radiologists don’t normally get taught through academia is the reading of normal cases”. [I02]* The difficulty with breast screening, as opposed to a diagnostic setting is that the prevalence of cancer is very low. So one might be reading 100 cases and find no cancers because the incidence is less than one in 100 of that screened cohort. To overcome these limitations of real life experience, radiologists discussed how ongoing self-training and education can improve their perception of both normal and abnormal breasts:

*Well, I do a lot of my own reading. I get involved … I go to talks, I go to workshops … I go online, we have our cancers [reports]. So there's a lot of material out there, and obviously you’ve got courses. I go the ‘Tabar’ screen reading course too. [I06]*

##### Systematic searching

The interviews revealed that there are similar viewing protocols for the display of mammograms and although there are differences from one place to the other, they tend to read the cases in a similar sequence, including the use of post-processing tools (such as magnification or changing the contrast threshold of the image). However, some radiologists believed other readers may skip some sequences such as the magnification in order to read faster. One concept that emerged from interviews was an emphasis on the importance of following all protocol steps, using available additional tools like magnification and not taking shortcuts:

*I think if you take shortcuts, you’ll miss things. So I think it’s a two-stage process: there’s the overall gestalt, but then there’s the process to go through the fine yarns. [I11]*

#### Feedback and expertise

Radiologists also discussed the impact of feedback for expertise development in breast radiology. They talked about different feedback procedures which can be employed for this purpose: feedback from following their previously reported cases, feedback mechanisms available through BreastScreen Australia, and feedback gained from educational and professional development activities.

##### General feedback on reported cases

The interviews revealed the importance of following up previously reported cases as a quality assurance mechanism for radiologists’ ability to distinguish abnormal and normal. When radiologists recommend alternative or additional forms of medical imaging such as ultrasound or Magnetic Resonance Imaging (MRI), or a suspicious lesion is present and the case requires biopsy, they have the opportunity to gain feedback through clinical audits. Radiologists believed this allows them to personally validate their original assessment as to whether a lesion was benign or malignant:

*In breast imaging, you do the biopsy, and the pathologist who gives a final answer. So it’s the gold standard, to correlate what you thought it was with what the pathologists say. [I01]*

Most participants believed that while the number of reads extensively impacts how a radiologist performs, getting feedback on what he/she has done before may be equally as significant. If the files of assessment clinic decisions and related pathology reports are available, breast radiologists acknowledged that they can review the records of missed cancers by accessing outcomes. Thus, an ideal feedback-expertise loop was described as where radiologists see a lot of cases and then review their decisions through the clinic or cytology records to inform their decision making process.

*If you never learn during those years of experience, then basically, you never improve and you never get better. So I think from my own personal point of view, it’s been more important getting feedback. So I think the feedback is a critical part of the learning process. [I17]*

Working in assessment clinics, where recalled women present for additional imaging, biopsy or clinical assessment were also recognised as important ways to attain feedback on what radiologists have reported. Within the interviews, the breast was discussed as a variable organ (in terms of shape, size and density) and mammograms were acknowledged as a two-dimensional view of a highly mobile three dimensional structure. Assessment clinic work provides the radiologist with a real-life understanding of the abnormalities and their difference with normal cases through patient interaction. “*Assessment clinic refines [radiologists’] selection criteria to better select a cancer … and sometimes to select a lesion they will never recall because it looks like normal breast tissue, but it is actually a cancer”. [I02]*

##### BreastScreen feedback loop

Interviewees discussed the feedback system at BreastScreen Australia as a review and self-learning process. Firstly, the system of double or arbitrating reading provided radiologists with good feedback about their performance by same colleagues. Secondly, the monthly process of sending the results of all biopsies that have been conducted to the radiologists was considered very helpful. Radiologists had access to this list through BreastScreen Information System (BIS) and spoke of being able to scroll through recalled lists of assessment clinic and the pathology results. Moreover, every three months, each radiologist is provided with an individual report of their performance related to correct decisions:

“*It is performance reading versus clinical outcome, and that’s a report generated for each radiologist with the number of their reads, their recall rate, the number of cancers they have diagnosed, and the number of cancer they have missed”. [I02]*

*Feedback is very, very important … One of the reasons I like working in BreastScreen is that the feedback and the clinical audit is mandated. [I11]*

BreastScreen Australia has a compulsory program for new breast radiologists to enable them to develop expertise via a mentoring and auditing system. The policy mandates that new radiologists are required to do 2,000 shadow reads before they can formally start reporting mammograms autonomously. This means that they read under the same condition as a normal radiologist, with the same list of patients however a senior radiologist will review the concordant and the discordant cases with the junior radiologist and give him/her feedback on missed cancers and incorrect recalls. The radiologists in our study that had work links to BreastScreen considered this program to be highly beneficial:

*The best way to learn radiology is with somebody sitting with you, and showing you how it works. So the push by universities to go onto virtual lectures, in my mind is crap. You cannot teach somebody how to do anything with the hands or their mind in a virtual environment … [What should be encouraged is], face-to-face and hands-on feedback. [I15]*

A number of radiologists actively sought continuing professional development (CPD) activities with an embedded feedback mechanisms to enhance their performance. In Australia, the most attended educational activity was identified as the BreastScreen Reader Assessment Strategy (BREAST), which uses enriched test sets delivered via an on-line platform to assess radiologists’ performance and provide direct feedback in real time [37]. Although BREAST is not intrinsically linked to BreastScreen, all participating radiologists in this study who were also BreastScreen readers had used this self-directed performance tutorial to evaluate their expertise. *“[These tutorials] facilitate feedback loop, [and] the quality improvement loop. You do a Quality Assurance [QA] activity to identify a problem. You develop a Quality Improvement Plan [QIP]. Then you do QA again, and you assess it to see where the QIP is fixed up”. [I11]*

### Role of information and communication technologies (ICT)

This section presents the views of participating radiologists about the role of ICT in their work environments as the ICT was identified in the literature as a core facilitator or constraint to building social networks [38]. The responses given by the radiologists related mainly to their daily functional tasks in seeking communication, validation and feedback as well as their overall perception of the impact of ICT available to them in enabling social networks.

#### Essential radiology functions

Radiologists described ICT as essential to their working lives and spoke about the advantages ICT brought to them in conducting their daily functional tasks: the benefits of Picture Archiving and Communication System (PACS), Radiology Information System (RIS) and BreastScreen Information System (BIS) as well as access to online resources. Radiologists reflected upon the introduction of such ICT systems to their work and how it had transformed connectivity to cases and colleagues.

Radiologists conceived that ICT play a critical role in their work practices. In Australia, radiology work environments are mostly digital, and thus “*[ICT] is extremely important. It has revolutionised [radiologists’] working life and that is in both mammography and in general work … [So] it’s essential and it’s normal activity now”. [I11]*. When we asked radiologists about the role of ICT in undertaking their functional tasks and facilitating interaction between colleagues, they strongly believed that current technologies had a significant impact upon their workflow and the quality of work when compared to the past due to the accessibility of past cases or ability to transfer images to colleagues. They were positive about the way ICT facilitated the access to feedback for radiologists who wish to review their previous reports and clinical audits:

*With the digital cases, if you just want to look at a study from two years ago, there’s a button and there it is. So the PACS system, it’s fantastic. [I04]*

However, a number of radiologists mentioned that some PACS systems were badly implemented in their workplaces. They believed that “*badly implemented technology is much worse than no technology” [I14]* because this may create negative effects on their daily tasks, and potentially their performance and patient care, by introducing frustrations about viewing or transferring cases:

*[BreastScreen] have a separate PACS. Our [private] PACS is not very mammogram-friendly … So I do see that difference between [good PACS and bad PACS] when I go and read in BreastScreen [I16]*

##### Digital libraries

The digital world has changed the way the radiology workforce operates and the way that radiologists are searching for information related to work practices. According to the interviews, radiologists widely use digital sources of information such as search engines, digital libraries and medical websites. Radiologists regularly accessed digital libraries for radiology e-books and associated research journal articles, used decision support systems such as imaging pathways and accessed radiological and pathological pictorial libraries and data sets. Whether radiologists are using digital sources for educational and research purposes or solving daily job-related problems, we found that they mostly prefer non-relational sources (online databases) over relational sources (e.g. professional online forums):

*If I want to look up something I would mainly use Google, ClinicalKey and STATdx ─ it’s a resource for radiologists about differential diagnoses and a lot of medical conditions. So it’s very specific for radiologists for finding information about medical stuff. [I12]*

*There’s a lot of access to things like applications if you’re interested. PubMed or whatever. Or if there’s an interesting pathology brought up, you can always quickly go and have a look online at some article regarding that. [I17]*

#### Social/transportable workplaces

ICT also facilitated professional networking among radiologists and other clinicians. Our findings support the notion that ICT has changed the way radiologists, as medical practitioners, can interact, communicate and share information. The interviews revealed that it is common for radiologists to take advantage of tele-radiology for the transmission of radiological images from one workstation to another and hence transport their work – this created a mobile working and social environment. New technologies were found to facilitate radiologists’ networking to a degree that sometimes they do not essentially need to be present at the patient’s location to be able to interact with other clinicians in conducting procedures, and this was especially helpful for patients in rural locations:

*For the majority of my working life, I’ve been in individual sites where it was analogue-based. You couldn’t ask someone to look at the film because it’s on the light box. Now, it’s fantastic. On our PACS, we have a message box. And we can click on the key to send the extension number to our colleague who might be a hundred miles away. Now when the doctors ring up, the first thing you do is you call up the images on the PACS. So that really does facilitate review of the consultation you’re having with your referring doctors. [I11]*

*One day, I had the clinic in [location 200 km away] but I couldn’t go there, and I decided to do the clinic [from my home town] because I have access to the clinic cases through the BIS and the PACS. I was on the phone, and the radiographer was doing the mammographic workup … and the breast physician was doing the ultrasound … Everything was hanging [being viewed] on the PACS. [I02]*

When requesting a second opinion or advice, telephone and communication tools embedded within the PACS or RIS were the preferred standard technologies for radiologists. Our interviewees generally reserved email communications for special occasions rather than part of their daily information sharing. However, there were some technological and ethical barriers which may hinder and prevent breast radiology networking as noted by the participants. From the technical perspective, sending the images to a colleague for reviewing and providing second opinion requires DICOM formatting and “*we tend to be looking at very subtle things, and you really need a 5-megapixel screen to see that”. [109]* From an ethical perspective, the radiologists needed to consider the security of network matters and most of the conventional social networking sites and emails on public domains cannot be used for such purposes because of the privacy concerns:

*I don’t like the idea of emailing a patient’s personal details. In fact, my preferred way is internal RIS system with my colleagues. Directly by phone, I find that a more secure way, rather than having a patient’s personal details on email. So it’s rare that I’ll use it. [I10]*

## Discussion

**Fig. 1:**
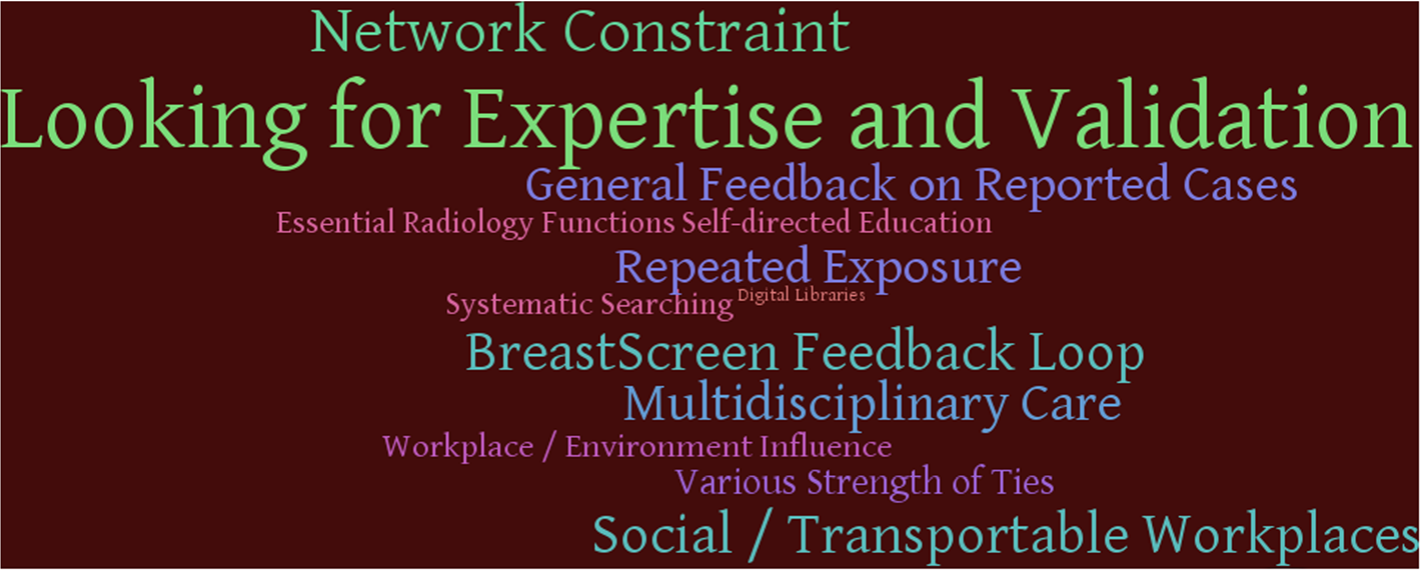
Word Cloud model of the concepts emerged from the interviews

Figure 1 shows a visual model of the strength of the concepts emerged from the interviews, where the size of each concept is correlated with the frequency of disclosing that concept by radiologists or the strength of conviction of the concept. Most radiologists interviewed believed that there is link between individual characteristics, social networks and level of ICT use, and combined in a number of ways, these all affect the performance of a breast radiologist. “*They all are intimately related to enhance performance. It is probably hard to separate into its components because we [radiologists] use all of those variables”. [I03]* Personal attributes such as the number of mammograms reads per week and the initiative and availability to follow up your own cases as well as professional networks and feedback from other radiologists and clinicians were all seen to impact positively upon performance. “*Where I’m learning most from, about reading mammograms, is from individual interactions. So, it’s the social network in the department, getting feedback from others in the department. It’s individual as well, following up my own cases, the feedback from my own cases”. [I12]* Moreover, ICTs were used to facilitate the feedback loop and enhance professional learning, which are required for improved performance. As one participant stated:

*I think the more years of experience you have, the more attuned your eyes become to looking at mammograms. But I think without feedback, and without other people telling you what they think of cases, and not consulting with other people, you can often do a large volume but you might not actually improve your skills. And like I said, without the PACS system, you wouldn’t have access to databases, extra cases for learning. So I think all three are interlinked. [I16]*

Most of the radiologists in this study held the opinion that without having access to feedback about their decisions, one may read a large volume of mammograms but actually not improve their skills and performance. According to the radiologists, social networking and getting a second opinion or validation of decision from other radiologists, particularly those considered experts, along with seeking feedback from other clinicians such as pathologists or participating in multidisciplinary care teams enables tacit knowledge transfer and skills improvement which ultimately lead to better performance. Moreover, our findings show that the role of ICT are significant not only because they provide access to online resources and extra cases which are required for ongoing training but also because they ease social networking, images transfer, immediate/real time feedback and promote learning process among the profession.

Participants in this study recognised a positive impact of social networking in providing new information and feedback to breast radiologists, which is in accordance with what has been suggested in the social network literature [6, 7]. Radiologists in this study equated social networks to key players in their networks that were important to them for different reasons, such as helping to advance professionally, discussing difficult cases and providing dual diagnosis or care for patients. The results show that the medium of communication may vary, for example in-person and telephone are more common than email.

Radiologists believed that functional diversity in their contacts (for example, direct communication and discussion with pathologists and other specialists, participation in MDT meetings) is important for gaining new knowledge and improving performance. However, the results from the interviews reveal that geographical diversity of radiologists’ contacts are normally limited within the same practice/radiology group where they work due to ICT constraints such as PACS, although PACS and BIS allowed for the transfer of cases between practice sites. This restriction may lead to increasing number of structural holes in radiologists’ professional network which ultimately decreases efficiency of interactions and redundancy of useful information provided to a radiologist. The strength of ties in terms of frequency of interactions with other radiologists varied for different radiologists participated in this study. We believe that this indicates an important area for further quantitative studies within the domain to evaluate the effects of social network measures on expertise development and performance levels.

## Conclusion

Previous studies on performance of breast radiologists show that observer performance is associated with some personal characteristics such as number of cases they read per week. In our study, while radiologists recognised the importance of reading high volumes and having exposure to a variety of cases in order to train and improve skills in reading mammograms, they also emphasised on the importance of feedback from their work systems, such as the BreastScreen reports. Additionally, radiologists identified social networking through expert seeking and validation, together with timely feedback on their decisions from other radiologists and specialists, was extremely important in developing expertise. Thus, through this study, performance can be viewed through the construct of constant independent and shared learning, either through platforms that provide feedback mechanisms or through professional networks mainly facilitated by ICT at the workplace.

An important implication from this study is that radiologists, as an example of knowledge intensive workers, recognised that they are highly dependent on informal knowledge sharing, feedback loops and new digital technologies to improve their skills and performance. These findings are in accordance with the previous research in the context of general practitioners [7]. In this regard, the concepts and ideas arise from this study can provide valuable insights in other areas of medical and health profession. We argue, from the findings of this study, that social learning is a valuable construct of expertise development and strategies such as facilitating efficient social interactions with diverse contacts and encouraging communication within work groups and also in the multidisciplinary sphere are essential in order to develop expertise and enhance performance in the workforce. Medical education programs need to appraise all types of learning in their curricula by giving attention to concepts of learning, learning by doing, apprenticeship learning, collaborative/social learning and case-based learning [21]. Moreover, medical practitioners and policy makers need to more broadly realise the value of social networks in professional expertise development.

## Additional file

Additional file 1: Appendix: Interview Questions. (DOCX 16 kb)

## Abbreviations

BIS: BreastScreen Information System
ICT: Information and communication technologies
PACS: Picture Archiving and Communication System
RIS: Radiology Information System
