# Supplementary material for: Social networks and expertise development for Australian breast radiologists
Source: BMC Health Serv Res. 2017 Feb 11;17:131. doi: 10.1186/s12913-016-1938-9 (PMC5307732; doi:10.1186/s12913-016-1938-9)
Supplement: Additional file 1: — Appendix: Interview Questions. (DOCX 16 kb) [file 12913_2016_1938_MOESM1_ESM.docx]

Appendix: Interview Questions

**SECTION** **A. Demographic and Workflow Questions**

| QUESTIONS |
| --- |
| 1. What is your specific job title? |
| 1. How many years have you been in current role/position? |
| 1. In which State do you work? |
| 1. When and from which medical school did you graduate? |
| 1. How many years have you been certified as a radiologist? |
| 1. Do you regularly read different medical images or only mammograms? |
| 1. How many years have you experience in reading mammograms? |
| 1. How many hours per week are you reading mammograms? |
| 1. How many mammograms per week are you reading? |
| 1. Can you please specify your workflow as a breast radiologist in Australia (e.g. what are your day-to-day tasks)? |
| 1. How are your daily tasks different from other radiologists’ ones? |
| 1. Is there any general problem(s) you can recognise in reading mammograms? |

**SECTION** **B. Exploration of Knowledge Acquisition and Expertise Development**

| QUESTIONS |
| --- |
| 1. Do you have any fellowship training (e.g. in breast imaging)? |
| 1. How often are you called to attend training or give training (e.g. to registrars)? |
| 1. What type of protocol/roadmap have you used/are you currently using for reading mammograms? |
| 1. Are these protocols applicable/useful in all reading cases? If not, what do you do alternatively? |
| 1. What expertise (i.e. special knowledge, skill or information) do you need for reading breast images which is not acquired through these protocols/ is not taught to registrars through academia? |

**SECTION C. Investigating the Influence of Social Networks**

| QUESTIONS |
| --- |
| 1. From time to time, you may come across difficulties in your practice as a breast radiologist, in particular in reading breast images. Do you seek help in professional matters from other people? |
| 1. Considering your daily tasks, how do you actually search for expertise in times of need? I.e. using formal organisational chart or informal network. |
| 1. Whom do you consider important for the provision of your medical practice and service? These people may be other radiologists or non-radiologist specialists (e.g. pathologists). |
| 1. In a monthly basis, how many people do you approach or you are approached by them to search for expertise? How frequent do you contact them? |
| 1. How diverse is your professional network in terms of geography and/or functionality? |
| 1. Do you think the team you are working with have a good map of each other’s skills and expertise (e.g. having a good idea of who knows what or who is an expert in what area)? |
| 1. In general, how do you think social networks affect a radiologist in terms of expertise development and performance? |

**SECTION D. Evaluation of Information and Communication Technologies (ICT) use***

| QUESTIONS |
| --- |
| 1. What mediums of ICT are used in your practice? Please specify which ones are most useful in context of your workflow. |
| 1. Do you usually use ICT for administrative and functional tasks (e.g. patients’ record keeping, clinical decision systems) or for social tasks (e.g. searching for expertise when needed using email communication)? |
| 1. Digital sources of information are classified as relational (e.g. professional online forums) and non-relational (e.g. online data bases). Which one(s) do you use for your professional tasks and how frequent? |
| 1. How important is ICT for transfer of information and communication among radiologists? |

**Information and Communication Technologies (ICT) are defined in this study as the technology that facilitates the transfer of information and communication to and from the radiologists.*

**SECTION E. Validation of the Theoretical Model**

| QUESTIONS |
| --- |
| 1. How do you see the relationship between these variables: social network properties, level of ICT use, personal attributes and reader performance. |
